# Myeloid‐Derived Suppressor Cells in Cancer: Mechanistic Insights and Targeted Therapeutic Innovations

**DOI:** 10.1002/mco2.70231

**Published:** 2025-05-31

**Authors:** Tianying Hu, Jianxue Zhai, Zhanda Yang, Jiajia Peng, Chuxuan Wang, Xinyao Liu, Yawen Li, Jiaqi Yao, Fengxi Chen, Haixia Li, Taixue An, Zongcai Liu, Haifang Wang

**Affiliations:** ^1^ Department of Laboratory Medicine Guangdong Provincial Key Laboratory of Precision Medical Diagnostics Guangdong Engineering and Technology Research Center for Rapid Diagnostic Biosensors Guangdong Provincial Key Laboratory of Single‐cell and Extracellular Vesicles Nanfang Hospital Southern Medical University Guangzhou China; ^2^ Department of Thoracic Surgery Nanfang Hospital Southern Medical University Guangzhou China; ^3^ The Laboratory of Endocrinology and Metabolism Guangzhou Women and Children's Medical Center Guangzhou Medical University Guangzhou China

**Keywords:** myeloid‐derived suppressor cells (MDSCs), MDSC phenotype, tumor microenvironment (TME), tumor‐promoting mechanisms, biomarker potential, therapeutic strategy

## Abstract

Myeloid‐derived suppressor cells (MDSCs) are a heterogeneous population of immature myeloid cells that expand aberrantly in cancer and exhibit potent immunosuppressive properties. They contribute to tumor progression through both immunological and nonimmunological mechanisms. Immunologically, MDSCs suppress antitumor responses by inhibiting effector cells such as T cells and NK cells, facilitating immune evasion. Nonimmunologically, they promote tumor growth and metastasis through processes such as the epithelial‒mesenchymal transition, angiogenesis, and premetastatic niche formation. MDSC accumulation is closely linked to accelerated tumor progression, including resistance to both immunotherapies and conventional treatments, making these cells critical therapeutic targets. Clinical studies have demonstrated the potential of MDSC‐targeted strategies to improve treatment efficacy. However, challenges remain in achieving specificity and effectiveness in MDSC‐targeted therapies, emphasizing the need for a deeper understanding of their biology. This review summarizes the origin, classification, and biological characteristics of MDSCs, their dual roles in tumor progression, and their clinical significance. We also discuss recent advances in clinical and preclinical studies, including both traditional targeted therapies and emerging innovative strategies. By integrating current findings, we aim to provide a comprehensive perspective on the role of MDSCs in cancer and valuable insights for advancing cancer treatment and drug development.

## Graphical abstract

This review summarizes the origin and biological characteristics of MDSCs, focusing on their immunological and nonimmunological roles in tumor progression. It also discusses the prognostic value of MDSCs and the latest advancements in clinical trials, highlighting the potential of emerging therapeutic strategies for cancer treatment and drug development.

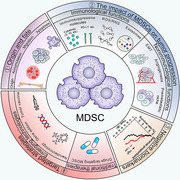

## 1 Introduction

The study of myeloid‐derived suppressor cells (MDSCs) began in the 1970s–1980s, when early research revealed that coculturing activated T cells with bone marrow cells suppressed T‐cell activity [1, 2]. At this stage, the focus of research was on identifying and understanding the role of immunosuppressive cells within the tumor microenvironment, although the exact nature of these cells remained unclear [3, 4, 5]. As research progressed into the early 2000s, a population of immature myeloid cells (IMCs) was shown to accumulate in the blood and tumor tissues of cancer patients, where they contributed to systemic immune suppression [6, 7, 8]. This accumulation correlated with both compromised immune surveillance and accelerated tumor progression [9]. These cells, initially referred to as myeloid suppressor cells (MSCs) or IMCs, were later recognized for their immunosuppressive functions [6, 10, 11]. MDSCs are a heterogeneous population of IMCs that are primarily composed of precursors of macrophages (Mφs), neutrophils, and dendritic cells (DCs), which accumulate in pathological conditions such as cancer [12]. The term MDSCs was formally introduced in 2007 to describe this heterogeneous population of IMCs that accumulate in pathological conditions, particularly in cancer [12]. Since then, research has increasingly focused on understanding the mechanisms by which MDSCs mediate immune suppression and promote tumor progression [10, 11].

In general, MDSCs play a pivotal role in tumor progression through their immunosuppressive functions [13]. During tumorigenesis, tumor‐derived cytokines and chemokines disrupt hematopoietic stem cell differentiation, leading to the abnormal proliferation of immature bone marrow cells [14, 15, 16]. These cells are characterized by their ability to suppress T cells, NK cells, and DCs through the secretion of immunosuppressive molecules, reactive oxygen and nitrogen species (ROS/RNS), and exosome‐mediated immune evasion [17, 18, 19, 20]. Subsequent studies have also shown that MDSCs promote tumor progression through various nonimmune mechanisms, such as inducing the epithelial‒mesenchymal transition (EMT), enhancing tumor angiogenesis, and establishing premetastatic niches, all of which facilitate tumor growth and metastasis [21, 22, 23].

In recent years, with advancements in research on MDSCs, their clinical importance has become increasingly evident [17]. Numerous studies have shown that MDSCs serve as crucial prognostic biomarkers for cancer progression and therapeutic effects, particularly in predicting the immune checkpoint inhibitor (ICI) response [24, 25, 26]. Additionally, MDSCs are emerging as promising therapeutic targets for cancer treatment [27, 28, 29]. However, despite the identification of several targeting strategies—such as depletion, functional inhibition, and differentiation induction—challenges such as low specificity and a lack of clinically effective drugs remain considerable hurdles [30].

**FIGURE 1:**
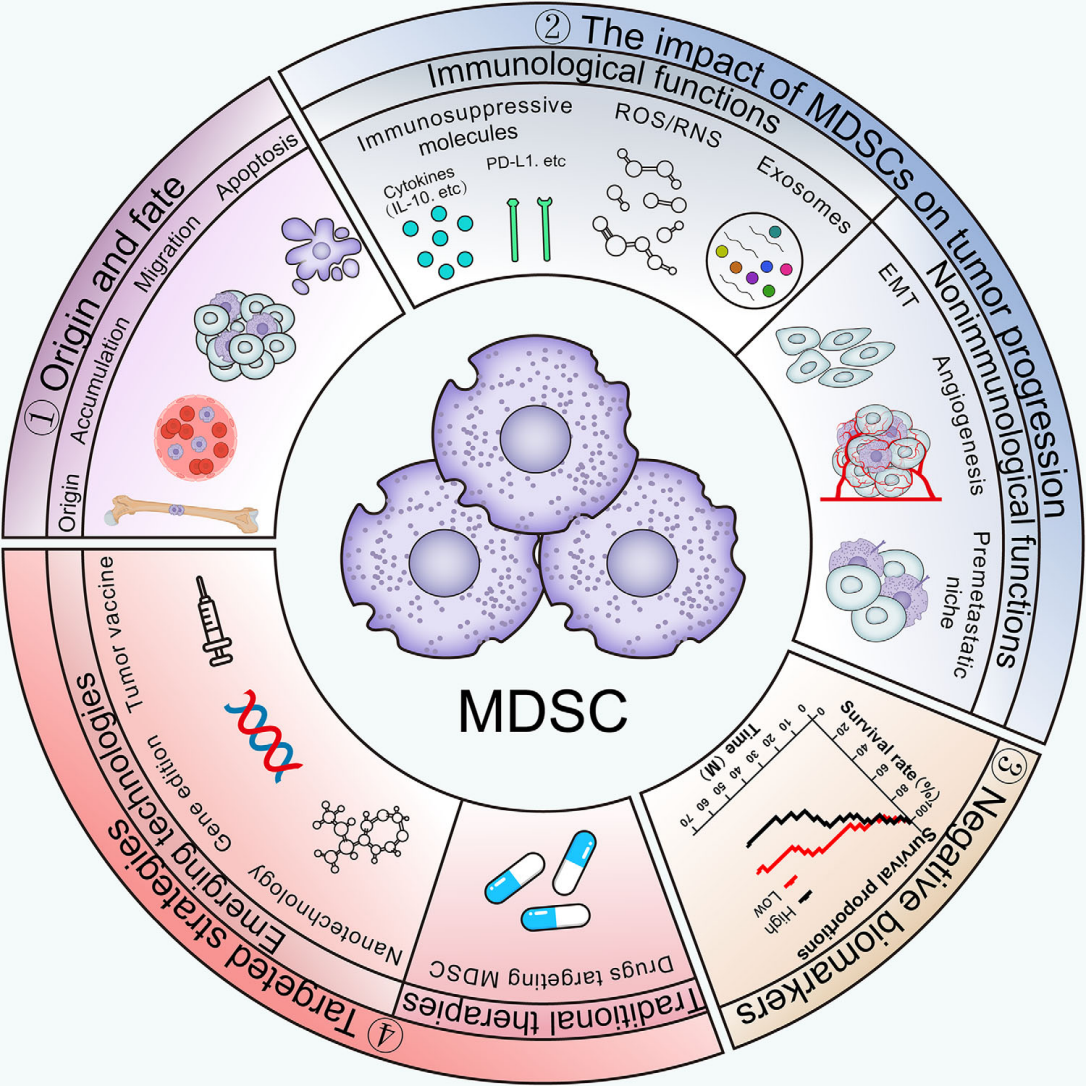
Summary of MDSCs in cancer. (A) The origin and fate of MDSCs. MDSCs are immunosuppressive cells derived from bone marrow. MDSCs expand in the bone marrow and traffic to the tumor microenvironment, where they ultimately undergo apoptosis. (B) The impact of MDSCs on tumor progression. MDSCs exert their functions through two primary pathways: ① Immunological functions: MDSCs suppress immune responses by expressing or secreting various immunosuppressive molecules, releasing ROS and RNS, and secreting exosomes, among other factors. ② Nonimmunological functions: MDSCs promote the EMT, enhance tumor angiogenesis, and facilitate the formation of premetastatic niches, thus driving tumor progression. (C) MDSCs are potential biomarkers for negative treatment responses and often correlated inversely with patient's survival. (D) Traditional and emerging therapies targeting MDSCs in cancer treatment. Innovative approaches, such as nanotechnology, can enhance treatment specificity compared with traditional MDSC‐targeted therapies, potentially improving therapeutic outcomes. *Abbreviations*: IL‐10, interleukin‐10; PD‐L1, programmed cell death ligand 1.

In this review, we systematically summarize the origin, classification, and biological functions of MDSCs; their dual roles in immunological and nonimmunological mechanisms in tumor progression; and their clinical significance. We also discuss recent advances in clinical and preclinical studies, including both traditional targeted therapies and emerging innovative strategies (Figure 1). By integrating current findings, we aim to provide a comprehensive perspective on the role of MDSCs in cancer and valuable insights for advancing cancer treatment and drug development.

## 2 Definition and Origin of MDSCs

### 2.1 Definition of MDSCs

**FIGURE 2:**
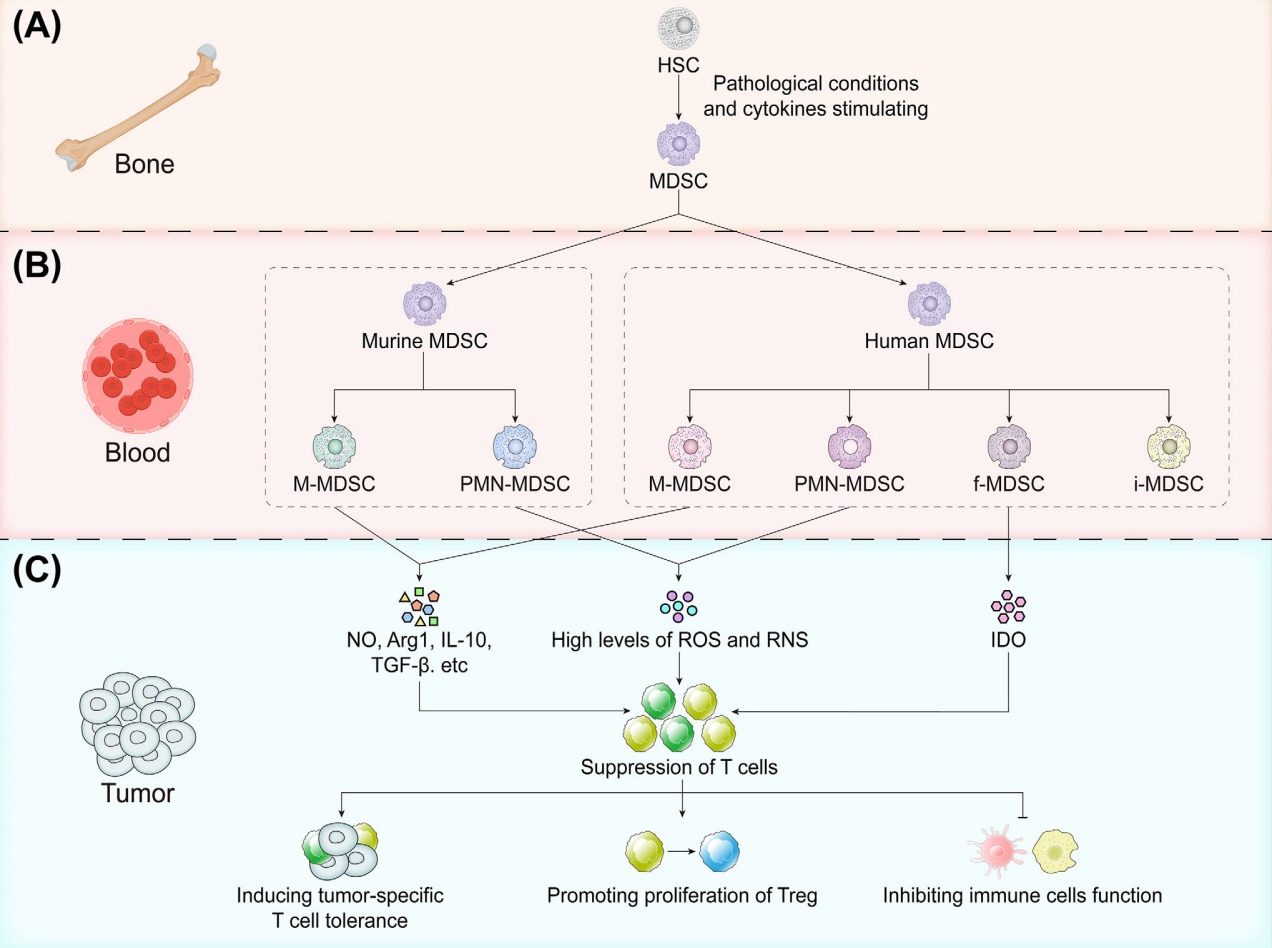
The origin and differentiation of MDSCs. (A) MDSCs originate from hematopoietic stem cells (HSCs) in the bone marrow. Under pathological conditions, various cytokines, including granulocyte‐macrophage colony‐stimulating factor (GM‐CSF), vascular endothelial growth factor (VEGF), interleukin‐1 beta (IL‐1β), interleukin‐6 (IL‐6), and IL‐10, drive MDSC production. (B) MDSCs differentiation in peripheral circulation. In humans, MDSCs are categorized into four main categories: M‐MDSCs (expressing CD14), PMN‐MDSCs (expressing CD15), i‐MDSCs, and f‐MDSCs. In mice, MDSCs are primarily divided into two subsets: M‐MDSCs and PMN‐MDSCs. (C) Functions of MDSC subsets within the tumor microenvironment. M‐MDSCs suppress T‐cell responses by secreting cytokines such as NO, Arg1, IL‐10, and TGF‐β, acting both in an antigen‐specific and nonspecific manner. PMN‐MDSCs inhibit T cells through elevated levels of ROS and RNS, which occurs via receptor–ligand binding. f‐MDSC inhibits T‐cell proliferation mainly via IDO secretion, thereby promoting the proliferation of Tregs. The function of i‐MDSCs remains to be fully elucidated. *Abbreviations*: HSC, hematopoietic stem cell, M‐MDSC, mononuclear MDSCs; PMN‐MDSC, granulocytic MDSC; f‐MDSC, fibrocytic MDSC; i‐MDSC, immature MDSC; NO, nitric oxide; Arg1, arginase 1; TGF‐β, transforming growth factor‐β; IDO, indoleamine 2,3‐dioxygenase; Treg, regulatory T‐cell.

MDSCs are a highly heterogeneous group of IMCs, which include immature myeloid progenitor cells and precursors to granulocytes, Mφs, and DCs [12]. Although MDSCs are a mixture of bone marrow cells with phenotypes such as granulocytes and mononuclear cells, their phenotypic markers remain unstandardized and have not been clearly verified [31]. This limited information is primarily because myeloid cells themselves have significant differences in their differentiation trajectories, and specific cell surface markers for MDSCs derived from DCs, Mφs, or monocytes are lacking. The current classification method still relies on morphology and surface markers to categorize MDSCs into subgroups (Figure 2).

In mice, the MDSC phenotype is identified by the expression of the myeloid differentiation antigens Gr‐1 and CD11b, defined as CD11b^+^Gr‐1^+^, with Gr‐1^+^ cells further divided into two main subtypes, Ly6G^+^ and Ly6C^+^, which are mouse‐specific markers and do not exist in humans. Based on the differences in Ly6G and Ly6C expression, mouse MDSCs can be divided into mononuclear MDSCs (monocytic MDSCs or M‐MDSCs) and granulocytic MDSCs (polymorphonuclear MDSCs, PMN‐MDSCs, or G‐MDSCs) [11, 23, 32].

In mice, PMN‐MDSCs are defined as CD11b^+^Ly6G^high^Ly6C^low^ cells, whose immunosuppressive mechanism relies mainly on high levels of ROS and RNS to inhibit T‐cell function [11]. Due to the high instability of ROS, which remain active for only a short time, close cell‐to‐cell proximity is required for PMN‐MDSCs to suppress T cells [11, 33]. Overall, the immunosuppressive function of PMN‐MDSCs is relatively weak, and the ultimate result of immunosuppression is mostly the induction of tumor‐specific T‐cell tolerance in tumors [23, 34–36].

The M‐MDSC subset is defined as CD11b^+^Ly6G^low^Ly6C^high^ cells, which mainly suppress T‐cell responses in an antigen‐specific and nonspecific manner by producing nitric oxide (NO), arginase 1 (Arg1), interleukin‐10 (IL‐10), and transforming growth factor‐β (TGF‐β) [11, 34]. Therefore, M‐MDSCs do not need close contact with T cells to exert an immunosuppressive effect. The suppressive activity of M‐MDSCs also depends on signal transducer and activator of transcription 1 (STAT1) and inducible NO synthase (iNOS), increasing intracellular NO levels while reducing ROS levels to thereby inhibit the differentiation process of IMCs into DCs and Mφs [23, 35, 36]. Additionally, M‐MDSCs are involved in tumor immune evasion. Tumor cells can promote the rapid differentiation of M‐MDSCs into tumor‐associated macrophages (TAMs) by downregulating the activity of signal transducer and activator of transcription 3 (STAT3), thus facilitating immune evasion [37, 38].

In humans, MDSCs primarily express the myeloid markers CD11b and CD33, as they lack a Gr‐1 homologous gene. They are typically categorized into two major subtypes: mononuclear cells expressing CD14 (monocytic‐like cells, M‐MDSCs, with the CD14^+^CD15^−^CD11b^+^CD33^+^HLA‐DR^−^Lin^−^ phenotype) and granulocytic cells expressing CD15 (granulocytic or polymorphonuclear‐like cells, G/PMN‐MDSCs with the CD15^+^CD14^−^CD11b^+^CD33^+^HLA‐DR^−^Lin^−^ phenotype) [31]. Additionally, other subgroups have been identified, including immature MDSCs (i‐MDSCs) and fibrocytic MDSCs (f‐MDSCs). i‐MDSCs are characterized by the surface markers CD45^+^LIN^−^HLA‐DR^−/low^CD11b^+^CD16^−^CD14^−^CD15^−^CD33^+^, although their exact function remains unclear [39]. Current research suggests that i‐MDSCs may promote intestinal tumor development by inducing receptor‐interacting protein kinase 3 expression [23, 39]. The f‐MDSC subset expresses CD33^+^IL‐4Rα^+^ and primarily exerts its immunosuppressive effects through the secretion of indoleamine 2,3‐dioxygenase (IDO), which inhibits T‐cell proliferation and supports regulatory T‐cell (Treg) expansion [23, 40].

### 2.2 The Fate of MDSCs: From Origin to Apoptosis

MDSCs include myeloid progenitor cells and IMCs. Under normal conditions, IMCs can rapidly differentiate into mature granulocytes, Mφs, and DCs and then enter the corresponding tissues and organs to perform their normal immune functions [41, 42, 43, 44]. However, under pathological conditions such as infections, chronic inflammation, and tumors, the maturation of myeloid progenitor cells is hampered, and these cells remain at various stages of differentiation, becoming MDSCs with immunosuppressive functions [20, 45, 46].

The survival or death of MDSCs is determined not only by the specific signaling molecules on MDSCs but also by the inflammatory environment induced by tumor cells and immune cells [47, 48]. The expression of proteins involved in signaling pathway activation, such as myeloid cell leukemia‐1, tumor necrosis factor receptor‐2 (TNFR2), cellular FLICE (FADD‐like IL‐1β‐converting enzyme)‐inhibitory protein, and IL‐4 receptor alpha (IL‐4Rα) within MDSCs, or a reduction in interferon regulatory factor‐8 expression, can inhibit apoptosis and thereby prolong MDSC survival [38, 45].

MDSC recruitment and accumulation are driven mainly by the binding of various growth factors and cytokines secreted by tumor cells and immune cells to corresponding receptors on the MDSC surface. For example, granulocyte‐macrophage colony‐stimulating factor (GM‐CSF) and granulocyte colony‐stimulating factor (G‐CSF) can activate the JAK/STAT1 and JAK/STAT3 signaling pathways to promote the recruitment of MDSCs [23, 36, 49, 50]. The expression of chemokine receptors (CXCR1/2, CCR2, etc.) and corresponding chemokines (CXCL1/2/5, CCL2, etc.) also plays a role in promoting their recruitment [51, 52, 53]. In addition, a team from Fudan University identified the molecule CD300ld, which induces STAT3 phosphorylation and then upregulates S100A8 and S100A9 expression, ultimately mediating the migration and recruitment of PMN‐MDSCs [54]. Interestingly, biorhythms have also been found to influence the accumulation of MDSCs in tumors [55].

Research has shown that MDSCs have a significantly shorter survival time than their corresponding monocytes and granulocytes in nontumor‐bearing mice, primarily because of changes in the expression of TNF‐related apoptosis‐inducing ligand receptors triggered by endoplasmic reticulum stress (ERS) [56]. Additionally, MDSC apoptosis is reported to be mediated by the upregulation of C/EBP homologous protein and death receptor 5 as a result of prolonged ERS [56].

## 3 The Role of MDSCs in Tumor Progression

**FIGURE 3:**
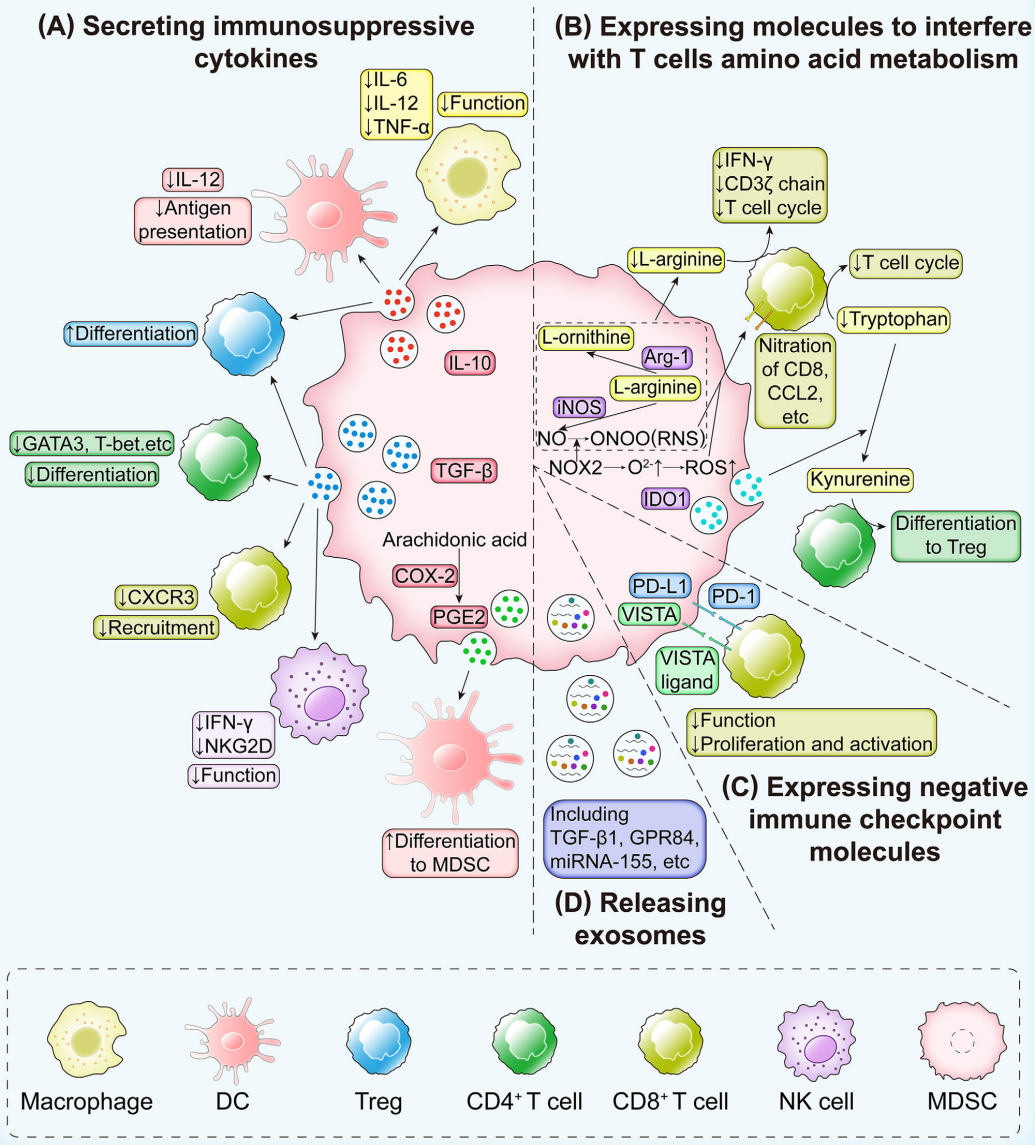
Immunosuppressive effect of MDSCs in tumor progression. (A) Secretion of immunosuppressive molecules. MDSCs exert their immunosuppressive effects through secreting a variety of cytokines and enzymes. For instance, IL‐10 secreted by MDSCs suppresses the production of cytokines such as IL‐12, which in turn inhibits the function of macrophages and DCs. TGF‐β released by MDSCs impedes T‐cell differentiation and recruitment, thereby dampening immune responses. Additionally, COX‐2 overexpression promotes PGE2 release, and thus induces DCs to redifferentiate into MDSCs, and finally inhibiting tumor cell apoptosis. (B) Metabolic disruption of T cells. MDSCs interfere with T‐cell metabolism by overexpressing Arg1, iNOS, and IDO1. These enzymes deplete key nutrients like l‐arginine and tryptophan, blocking the T‐cell cycle, reducing CD3ζ chain expression, and nitrating cell surface molecules such as CD8 and CCL2. Additionally, kynurenine generated via the IDO1 pathway promotes the naive T cells differentiating into Treg cells. (C) Expression of immune checkpoint. MDSCs express immune checkpoint molecules like PD‐L1 and VISTA, which interact with PD‐1 and VISTA receptors on T cells, leading to T‐cell suppression. (D) Exosome‐mediated immunosuppression. MDSCs release exosomes containing TGF‐β1, S100A8/9, miRNAs, and other molecules into the tumor microenvironment. These exosomes modulate the local immune response to exert immunosuppressive effects. *Abbreviations*: IL‐12, interleukin‐12; TNF‐α, tumor necrosis factor‐alpha; GATA‐3, GATA binding protein 3; CXCR3, receptor for CXC chemokine receptor 3; IFN‐γ, interferon‐γ; NKG2D, NK receptor group 2 member D; COX‐2, cyclooxygenase 2; PGE2, prostaglandin E2; PD‐1, programmed cell death protein 1; VISTA, V‐domain Ig suppressor of T‐cell activation; GPR84, G‐protein‐coupled receptor 84.

**FIGURE 4:**
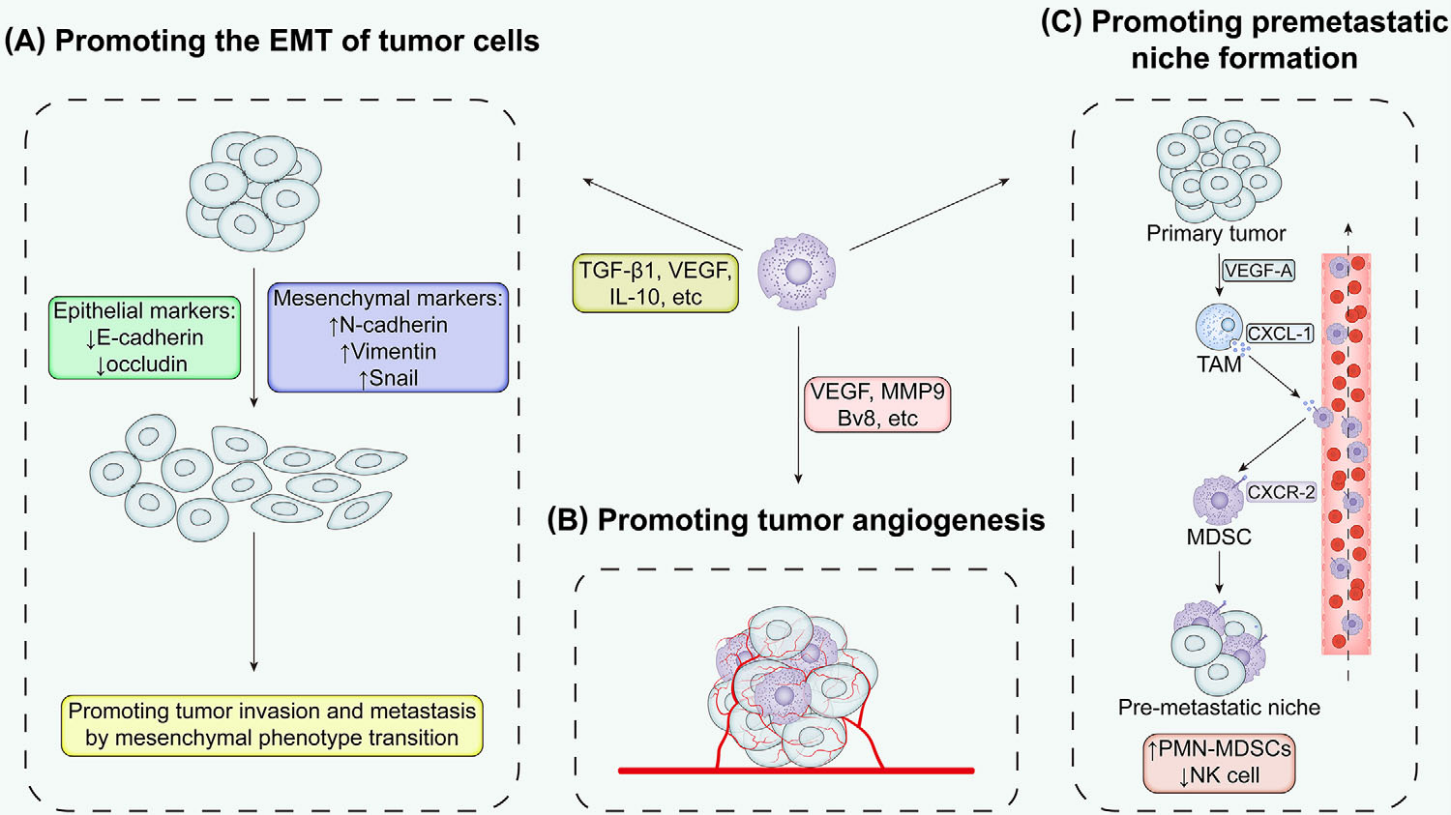
Nonimmunological functions of MDSCs in tumor progression. (A) Promoting the EMT of tumor cells. MDSCs secrete cytokines such as TGF‐β1, VEGF, and IL‐10, which upregulate mesenchymal markers in tumor cells and drive their EMT. (B) Promoting tumor angiogenesis. MDSC‐derived factors VEGF, Bv8, and MMP9 actively contribute to tumor angiogenesis, which support tumor growth and metastasis. (C) Promoting premetastatic niche formation. MDSCs are involved in the formation of the premetastatic niche. For example, in colorectal cancer liver metastasis models, VEGF‐stimulated tumor‐associated macrophages (TAMs) secrete CXCL1 to recruit CXCR2^+^ MDSCs to the premetastatic liver, creating an immunosuppressive environment. This MDSCs accumulation results in NK cells depletion, consequently impairing antitumor immunity. *Abbreviations*: MMP9, matrix metalloproteinase 9; Bv8, prokineticin 2.

The text above discusses the immunosuppressive mechanisms of PMN‐MDSCs and M‐MDSCs. Numerous studies have highlighted the proliferation of MDSCs across a range of human tumors, including cutaneous melanoma, hepatocellular carcinoma, breast cancer, prostate cancer, and lung cancer [11]. Clinical trials and existing findings indicate that the roles of MDSCs in tumor progression can be categorized into immune‐related and nonimmune‐related functions. The former involves the secretion of immunosuppressive cytokines, interference with T‐cell amino acid metabolism, the expression of negative immune checkpoint molecules in the tumor, and the release of exosomes (Figure 3); the latter involves promoting the EMT in tumor cells, facilitating tumor angiogenesis, and promoting the formation of premetastatic niches (Figure 4). This section discusses these functions in detail.

### 3.1 Immunological Role

#### 3.1.1 Secretion of Immunosuppressive Cytokines

##### 3.1.1.1 Interleukin‐10

Recent studies have revealed a positive correlation between serum IL‐10 levels and M‐MDSC abundance in patients with metastatic melanoma [57]. IL‐10, which is predominantly secreted by MDSCs within some tumors, plays a pivotal role in shaping the phenotype and functionality of MDSCs, highlighting the intricate crosstalk between the two entities [58]. IL‐10 is released by MDSCs and exerts immunosuppressive effects on tumors in multiple ways, such as downregulating IL‐6, IL‐12, and TNF‐α expression in Mφs [59, 60]. Furthermore, IL‐10 inhibits IL‐12 production in DCs, impairing their antigen presentation and migration [61]. Recent studies have shown that naive Foxp3^+^ T cells differentiate into Tregs in the presence of IL‐10 and TGF‐β [62]. Notably, in murine models, the proliferation of chimeric antigen receptor‐T (CAR‐T) cells is significantly inhibited by CD11b^+^Gr1^+^ bone MDSCs when they are cocultured. Conversely, the administration of an anti‐IL‐10 antibody markedly increased the population of CD8^+^ T cells [63]. In the above process, IL‐10 release is associated with the JAK/STAT signaling pathway [64]. These studies underscore the strong association between elevated IL‐10 levels and MDSC‐induced immunosuppressive effects on tumors.

##### 3.1.1.2 Transforming Growth Factor‐β

As a well‐known immunosuppressive molecule, TGF‐β inhibits the body's antitumor immune response in inflammatory and tumor environments in various ways, including inhibiting the activities of cytotoxic T lymphocytes (CTLs), T follicular helper cells, and NK cells; preventing the differentiation of CD4^+^ T cells into Th1 and Th2 cells; and inducing Treg and Th17 production [65, 66, 67]. TGF‐β affects T‐cell differentiation by inhibiting the expression of T‐bet and GATA‐3 (key regulators of Th1 and Th2 differentiation, respectively), thereby blocking their differentiation [68, 69]. Regarding the inhibition of T‐cell function, as early as 2012, some scientists found that CD14^+^ HLA‐DR^−^ cells secreted large amounts of TGF‐β while reducing the expression of interferon‐γ (IFN‐γ) [70]. In addition, TGF‐β limits the infiltration of peripheral CD8^+^ T cells into the tumor site by reducing the expression of CXC chemokine receptor 3 (CXCR3) on the membrane of CD8^+^ T cells. TGF‐β increases the activation threshold of intratumoral CD8^+^ T cells and significantly reduces their immune function [71]. In addition to exerting the functions mentioned above, TGF‐β affects MDSC production and differentiation. Gneo et al. reported that TGF‐β released by colorectal cancer cells activates the STAT3 pathway, which in turn induces the generation of M‐MDSCs [72, 73, 74]. In response to other immune cells, MDSCs cause a decrease in IFN‐γ expression in NK cells through the expression of membrane‐bound TGF‐β, along with the downregulation of membrane NK receptor group 2 member D (NKG2D) [75].

##### 3.1.1.3 Cyclooxygenase‐2 and Prostaglandin E2

Cyclooxygenase (COX) is classified into three isoforms, among which COX‐2 is expressed at a low or undetectable level in normal organisms, but it is highly expressed in a variety of precancerous lesions and cancers [76]. COX‐2 acts in the tumor microenvironment (TME) through a variety of mechanisms, such as inhibiting tumor cell apoptosis, promoting prostaglandin E2 (PGE2) release, and inducing IDO1 production [77]. MDSCs mediate tumor immunosuppression in melanoma through the expression of COX‐2, PGE2 and Arg1 [78]. High COX‐2 expression is significantly associated with lower tumor‐infiltrating lymphocyte levels. Furthermore, in triple‐negative breast cancer, elevated COX‐2 levels are linked to increased levels of ERS and autophagy markers, with tumor cells relying on this heightened stress response to sustain rapid growth [79]. Additionally, the PGE2 receptor 4 subtype (EP4) on MDSC membranes also binds to tumor cell‐expressed PGE2, a signaling pathway that promotes MDSC secretion of Arg1, thereby suppressing tumor immunity [80, 81]. Research indicates the existence of a positive feedback loop between PGE2 and COX‐2, redirecting DCs toward MDSC differentiation and contributing to the maintenance of MDSC functional stability [82].

#### 3.1.2 Expression of Molecules that Interfere with Amino Acid Metabolism in T Cells

##### 3.1.2.1 Arginase1

In 2005, Zea et al. [83] reported Arg1 overexpression in renal cancer. They found that increased Arg1 activity was restricted to the CD11b^+^CD14^−^CD15^+^ cell population in the peripheral blood of patients with metastatic renal cell carcinoma. High Arg activity was observed only in the mature CD11b^+^/Gr‐1^+^ subpopulation and not in immature CD11b^+^/Gr‐1^+^ myeloid cells or CD11b^−^/Gr‐1^+^ cells [84, 85]. High expression of Arg1 by MDSCs is regulated by Th2‐type cytokines such as IL‐4 and IL‐13 [86]. In mice, tumor‐associated myeloid cells highly express cationic amino acid transporter protein‐2B (CAT‐2B), which facilitates l‐arginine uptake into MDSC from the extracellular space [84]. Conversely, human MDSCs do not express CAT‐2B, implying that they do not take up arginine from the TME but instead release Arg1 into the TME through degranulation [87]. l‐Arginine is then catabolized into urea and l‐ornithine by Arg1, depleting essential amino acids from the TME, which are essential substrates for T‐cell activation [84]. The lack of l‐arginine reduces the expression of the CD3ζ chain, although the exact mechanism remains unclear; it may involve the specific inhibition of the translation of the CD3ζ chain by l‐arginine [88, 89]. Additionally, the expression of T‐cell cytokines such as IFN‐γ and IL‐5 is reduced, which may be related to the decreased mRNA expression caused by l‐arginine deficiency [89]. In addition to the effects mentioned above, arginine deficiency induces G0‒G1 phase arrest in the T‐cell cycle by blocking the upregulation of cyclin D3 and cyclin‐dependent kinase 4 (CDK4), significantly reducing retinoblastoma‐forming phosphorylation and thereby preventing the cellular transition from G1 phase to S phase [47].

##### 3.1.2.2 ROS and RNS

l‐Arginine is metabolized not only by Arg1, as described above, but also by iNOS, which converts l‐arginine to NO and l‐citrulline. iNOS expression is induced by Th1 cytokines such as IFN‐γ, TNF‐α, and IL‐1β. iNOS, once considered a hallmark of M1‐type Mφs, is also expressed in MDSCs [90]. A recent study highlighted the role of *Peptostreptococcus anaerobius*, a member of the oral microbiome, in promoting tumor progression. In colorectal cancer, *P. anaerobius* secretes the N‐acetylmuramoyl‐l‐alanine amidase (lytC_22) protein, which binds to the signaling lymphocytic activation molecule gene family (Slamf) 4 receptor on MDSCs. This interaction promotes Arg1 and iNOS expression in MDSCs, further increasing their immunosuppressive activity and contributing to tumor progression [91].

In addition to iNOS, MDSCs express nicotinamide adenine dinucleotide phosphate oxidase 2 (NOX2), which increases ROS levels in the form of superoxide (O^2−^). These ROS interact with NO to form peroxynitrite (ONOO), a potent RNS that further contributes to immune suppression [92]. Subsequently, RNS mediate CCL2 nitration, reducing the affinity between CCL2 and CCR2 on CD8^+^ T cells. This process results in the impaired recruitment of T cells into the tumor microenvironment and weakens antitumor immune responses [93]. Furthermore, ROS catalyze the nitration of CD8 molecules, blocking TCR/MHC–peptide interactions and further hindering T‐cell activation [94].

Notably, a positive feedback regulatory mechanism exists between MDSCs and ROS. Specifically, MDSCs promote an increase in ROS levels, and ROS can influence MDSC differentiation by regulating the expression of related genes. This regulation prevents MDSCs from differentiating into TAMs and DCs, which would otherwise support antitumor immunity [95]. For example, *H_2_O_2_ scavenging*—the process of reducing hydrogen peroxide levels using enzymes such as catalase—has been shown to promote the differentiation of IMCs into Mφs in tumor‐bearing mice. By lowering ROS levels, *H_2_O_2_ scavenging* alleviates the inhibitory effects on differentiation and promotes immune cell maturation, highlighting a potential therapeutic approach for modulating MDSC differentiation in tumors [96].

##### 3.1.2.3 Indoleamine 2,3‐Dioxygenase1

IDO1 is frequently overexpressed in various cancer types, where it exerts a potent immunosuppressive effect within the TME. The activation of STAT3 through phosphorylation further increases IDO expression, reinforcing the immunosuppressive landscape [97]. This enzyme catalyzes the hydrolysis of tryptophan to kynurenine, leading to a lack of tryptophan in the TME. This depletion, in turn, activates general regulatory inhibition of protein kinase 2 (GCN2), inducing the downregulation of the TCR ζ‐chain on the T‐cell membrane and subsequent cell cycle arrest [98]. Moreover, kynurenine promotes T‐cell differentiation into CD25^+^FoxP3^+^ Tregs by binding to the aromatic hydrocarbon receptor (AHR) on T cells [99].

Notably, crosstalk between MDSCs and IDO1 has been observed. Certain populations of MDSCs in the human body express IDO1, which, in turn, influences the recruitment and accumulation of MDSCs [100, 101]. In addition, studies have shown that in virus‐associated liver cancer, hepatitis Be antigen recruits M‐MDSCs and promotes their expansion. This process inhibits the ability of IDO1 to mediate CD8^+^ T‐cell responses in vitro, further highlighting the complex interactions between MDSCs and IDO1 in shaping the immune response in the TME [36].

#### 3.1.3 Expression of Negative Immune Checkpoint Molecules

##### 3.1.3.1 PD‐1/PD‐L1

ICIs, especially inhibitors of programmed cell death protein 1 (PD‐1) and its ligand PD‐L1, have shown marked therapeutic efficacy in cancer treatment [102]. Tumor cells achieve tumor immune escape by expressing PD‐L1, which binds to PD‐1 on the T‐cell membrane. Similarly, MDSCs inhibit T‐cell function by interacting with PD‐1 on T cells and simultaneously upregulating their own PD‐L1 expression [13, 103]. MDSC surface PD‐L1 expression is regulated by multiple pathways. For example, hypoxia leads to the significant upregulation of PD‐L1 on splenic MDSCs in tumor‐bearing mice, and this upregulation depends on hypoxia‐inducible factor‐1α (HIF‐1α), which regulates PD‐L1 expression by directly binding to the transcriptionally active hypoxia‐responsive element in the proximal promoter of PD‐L1 [104, 105]. In contrast, a subpopulation of MDSCs expressing G protein‐coupled receptor 84 (GPR84) exerts immunosuppressive effects by inhibiting PD‐L1 degradation in lysosomes [106].

##### 3.1.3.2 V‐Domain Ig Suppressor of T‐Cell Activation

The V‐domain Ig suppressor of T‐cell activation (VISTA) is a recently identified immunomodulatory molecule that has the characteristics of the B7 and CD28 immunomodulatory molecule families. VISTA can act as both a ligand and a receptor [107, 108]. Both mouse and human VISTA are expressed on lymphoid and myeloid cells, with the highest expression (as a receptor) on primitive CD4^+^ T cells. Human VISTA interacts with two identified binding chaperones with immunosuppressive functions, P‐selectin glycoprotein ligand‐1 (PSGL‐1) and V‐Set and immunoglobulin domain containing 3 (VSIG3), both of which suppress T‐cell function through mutual interaction. Specifically, VISTA binds to VSIG3 at normal physiological pH, whereas it binds to PSGL‐1 in acidic environments [109, 110, 111].

VISTA acts as a ligand on tumor cells and mediates negative immunomodulation by inhibiting CD4^+^ and CD8^+^ T‐cell activity and suppressing immune cell activity. When VISTA molecules act as receptors, they are highly expressed on MDSCs and significantly suppress immune responses [112]. MDSCs from patients with acute myeloid leukemia (AML) express significantly increased levels of VISTA, and knockdown of VISTA reduces the inhibition of CD8^+^ T‐cell activity by MDSCs in AML patients [113]. In the CT26 murine colon cancer model, tumor hypoxia drove VISTA expression via HIF‐1α, which was highest in CD11b^high^ Gr1^+^ MDSCs, and hypoxia‐induced VISTA expression in MDSCs contributed to its inhibition of T‐cell proliferation and activation [114]. Additionally, a lack of VISTA in the TME reduces STAT3 activation, decreases Arg1 expression and causes mitochondrial dysfunction in MDSCs [115].

#### 3.1.4 Release of Exosomes

Exosomes are extracellular vesicles (EVs) that are approximately 30–150 nm in diameter. Unlike ectosomes, exosomes are intraluminal vesicles formed by the invagination of the plasma membrane, and their release involves the fusion of multivesicular bodies with the plasma membrane. Exosomes carry a variety of cellular components, including DNA, RNA, lipids, metabolites, and both cytoplasmic and cell‐surface proteins. By transferring these specific components to recipient cells, exosomes play crucial roles in intercellular material transport and signaling [116, 117, 118, 119]. Similarly, exosomes released by MDSCs (MDSC‐derived exosomes) inherit the immunosuppressive, protumor growth, angiogenic, invasive, and metastatic capacities of their parental cells [120, 121]. For example, TGF‐β1 enriched in MDSC‐derived exosomes may induce Treg production and diminish the killing capacity of NK cells. In addition, miRNA‐155 is highly enriched in MDSC‐derived exosomes, and this RNA induces MDSC production by targeting Src homologous 2‐inositol phosphatase‐1 and thereby inducing MDSC expansion [122, 123]. In the spleens of animals treated with splenic MDSC‐derived exosomes, the number of M1‐type Mφs was significantly reduced, whereas the number of M2‐type Mφs was increased. Additionally, MDSC‐derived exosomes deplete CD8^+^ T cells in vivo, indicating their immunosuppressive role in the TME [124]. Recent studies have shown that MDSC‐produced GPR84‐positive exosomes are taken up by CD8^+^ T cells and then suppress T‐cell function by activating the p53‐mediated ROS‒DNA damage pathway [125].

### 3.2 Nonimmunological Functions

#### 3.2.1 Promotion of the EMT in Tumor Cells

Cells are able to transition between epithelial and mesenchymal states in a highly plastic and dynamic manner during fetal development. This process, known as the EMT, plays a critical role in cancer progression [23]. MDSCs are also reported to mediate the EMT of tumor cells in various cancers, resulting in the loss of differentiated epithelial characteristics and structural integrity. Tumor cells then acquire mesenchymal traits, which fundamentally change the morphology and movement of cells and thus promote tumor invasion and metastasis [126, 127, 128, 129].

In a 2019 study exploring the effects of MDSCs on the EMT in tumor cells, MDSCs were extracted from tumor‐bearing mice, and the expression of TGF‐β1, vascular endothelial growth factor (VEGF), and IL‐10 was detected, which further suggested that these cytokines play crucial roles in inducing the EMT [130]. Additionally, by coculturing breast cancer cells with MDSCs, researchers found that the expression of mesenchymal markers such as N‐cadherin, vimentin, and the transcription factor Snail is increased, whereas the expression of the epithelial markers E‐cadherin and occludin is decreased. Mechanistically, they reported that MDSCs induce the EMT in breast cancer cells by activating the PI3K/AKT/mTOR signaling pathway and increasing the expression of matrix metalloproteinases (MMPs), thus increasing the invasion and metastatic potential of breast cancer cells [131]. A recent study of the bone metastasis of prostate cancer revealed that disseminated tumor cells in the bone marrow microenvironment expressed bone marrow cell markers and that these fusion cells had an increased tumorigenic ability during the EMT [85].

#### 3.2.2 Promotion of Tumor Angiogenesis

Tumor angiogenesis is an important condition for tumor development, invasion, and metastasis. During tumor growth, especially solid tumor growth, the tumor microenvironment requires angiogenesis to provide adequate oxygen and nutrients and remove waste products, which are conducive to optimal tumor growth. MDSCs can promote tumor angiogenesis through multiple mechanisms during this process [132]. Yang et al. [133] confirmed that coinjection of Gr‐1^+^CD11b^+^ cells with tumor cells in a mouse tumor model can increase tumor angiogenesis and vascular maturation compared with the injection of tumor cells alone, and this group of cells mainly promotes angiogenesis by secreting MMP9 and VEGF. In addition, Gr‐1^+^CD11b^+^ cells integrated directly into the neovascular endothelium and significantly upregulated the expression of endothelial cell markers. Gr‐1^+^CD11b^+^ cells also promoted tumor angiogenesis through the G‐CSF/STAT3/prokineticin 2 (Bv8) axis, while anti‐BV8 treatment could significantly reduce the tumor volume and decrease the number of Gr‐1^+^CD11b cells and their activity, indicating the positive role of MDSCs in tumor angiogenesis [134, 135].

#### 3.2.3 Promotion of Premetastatic Niche Generation

Primary tumor‐derived components, tumor‐mobilized bone marrow‐derived cells, and the host's local stromal microenvironment (or future metastatic organ components) are the three major factors involved in the formation of premetastatic niches [136]. Tumor cell‐derived chemokines and cytokines recruit MDSCs, TAMs, Tregs, and tumor‐associated neutrophils to distant secondary sites, collectively shaping the premetastatic tumor niche [136, 137].

MDSCs have been shown to play a critical role in multiple types of tumor metastases. For example, MDSCs accumulate in the liver before colorectal cancer metastasis, and the number of MDSCs in the premetastatic niche is negatively correlated with the number of NK cells in patients with colorectal cancer, indicating favorable regulation of liver metastasis in these patients. In addition, glycolytic metabolism helps to regulate the recruitment of MDSCs in the microenvironment before metastasis, and increased numbers of MDSCs, in turn, are likely to facilitate the metastasis of tumor cells to the liver through HIF‐1α signaling [138].

Additionally, in a mouse model, VEGF‐A secreted by colorectal cancer cells stimulated the production of CXCL1 by TAM and recruited CXCR2^+^ MDSCs from the circulatory system into the premetastatic liver, further serving as support cells to maintain the survival of tumor cells with liver metastasis [139]. Interestingly, researchers have reported that chronic psychological stress can also promote the accumulation of CD11b^+^Gr‐1^+^ MDSCs in the lungs of patients with breast cancer, thus promoting the lung metastasis of breast cancer via a mechanism similar to that of the CXCL1/CXCR2 axis mentioned above [140].

Like premetastatic niches at distant sites, premetastatic lymph node niches are also found in patients with gynecological cancers. For example, the lymph nodes within the drainage area of high‐grade endometrial cancer contain many S100A8/A9 (calprotectin)‐positive MDSCs and CD163^+^ M2 Mφs, which diminish immune activity in this area [141].

In addition, the tumor microenvironment, which is involved in the formation of MDSCs, is associated with tumor recurrence [142]. For example, clinical studies have shown that despite radiofrequency ablation treatment for hepatocellular carcinoma, the recurrence rate remains high due to the accumulation of PMN‐MDSCs induced by the METTL1/TGF‐β2 mechanism in the post‐treatment cancerous tissue [143]. Thus, inhibiting the accumulation of MDSCs could reduce the likelihood of tumor recurrence [136].

## 4 MDSCs as Biomarkers of a Negative Treatment Response

ICIs targeting PD‐1 and CTLA‐4 have profound therapeutic effects in the clinic. However, the treatment response varies greatly from individual to individual, and an important aspect related to the difference in patients’ responses is the immunosuppressive effect caused by the TME [144, 145]. Research has shown that MDSCs, with their potent immunosuppressive activity, can diminish the efficacy of immunotherapy and reduce the survival times of patients with tumors [146]. Therefore, elucidating the clinical value of MDSCs in assessing patient prognosis and treatment efficacy holds profound importance.

Furthermore, elucidating the clinical value of MDSCs in immunotherapy is highly important, as the evidence suggests that MDSCs could also serve as predictive biomarkers for poor outcomes of immunotherapy across various cancers [35].

The evidence suggests that MDSCs can also serve as predictive biomarkers for a poor prognosis of various types of cancer [35]. For example, the frequency of MDSCs in the peripheral blood of melanoma patients who respond to ipilimumab (a CTLA‐4 monoclonal antibody) is lower than that in patients who do not respond [147, 148]. The levels of CD33^+^ MDSCs are elevated in cervical tumors. High levels of CD33^+^ MDSCs are significantly associated with shorter disease‐free survival (DFS) and overall survival (OS) of cervical cancer patients [149]. In a prospective study of metastatic breast cancer, high levels of CD14^+^ monocyte‐type MDSCs (Mo‐MDSCs) represented aggressive disease and worse outcomes for patients, and patients with high Mo‐MDSC levels experienced shorter survival [150]. Therefore, Mo‐MDSCs can be considered a reference for evaluating the possibility of breast cancer metastasis. In addition, the frequency of MDSCs increases with the tumor stage and the presence of lymph node and/or distant metastases, indicating a correlation with clinical cancer stage [17].

In assessing treatment efficacy, MDSCs can be used as a predictor of anti‐PD‐1 immunotherapy efficacy [151, 152]. For example, in patients with non‐small cell lung cancer (NSCLC), the ratio of Tregs to lectin‐type oxidized low‐density lipoprotein receptor 1 (Lox‐1) MDSCs was positively correlated with anti‐PD‐1 efficacy [153]. Youn et al. [154] showed that the ratio of NK cells to Lox‐1 PMN‐MDSCs was also a promising biomarker for PD‐1 antibody therapy. A low frequency of MDSCs in peripheral blood is associated with longer progression‐free survival and OS of NSCLC patients after PD‐1 treatment [92, 155].

Furthermore, MDSCs can also serve as biomarkers for evaluating the efficacy of radiotherapy. Partial radiotherapy can alter the abundance and immunosuppressive activity of MDSCs and ultimately the therapeutic effect [156]. Specifically, the depletion and expansion of MDSCs after radiotherapy are associated with preclinical tumor models and incomplete treatment responses in cancer patients [157]. Thus, MDSCs stand out as promising targets to improve the efficacy of radiotherapy treatment, as observed (at least in preclinical settings) via a variety of pharmacologic interventions or genetic approaches.

## 5 Traditional Targeted Therapeutic Approaches for MDSCs in Cancer

Previous studies have shown that MDSCs not only facilitate tumor immune evasion by inhibiting the functions of immune cells within the TME but also enhance tumor progression through various mechanisms, including the promotion of the tumor cell EMT, angiogenesis, and premetastatic niche formation. Consequently, targeting MDSCs has emerged as a promising strategy in cancer therapy [92]. Based on the characteristics and functions of MDSCs in tumor progression, therapeutic strategies can be generally classified into five categories: ① inhibiting MDSC proliferation and recruitment; ② promoting the differentiation of MDSCs into nonsuppressive immune cells; ③ promoting the apoptosis of MDSCs; ④ inhibiting MDSC metabolism; and ⑤ inhibiting the immunosuppressive function of MDSCs. The following sections elaborate on these strategies (Table 1).

**TABLE 1:** Clinical and preclinical studies of MDSC‐targeting strategies in cancer therapy.

Mechanism of action	Target	Drug name	Combination therapy	Indications	Type of trial	References
Inhibiting MDSC proliferation and recruitment	VEGF	Bevacizumab	Chemotherapy	Non‐small cell carcinoma	Clinical trial	[158]
		Endostar	Anti‐PD‐1 therapy	Lewis lung carcinoma	Animal experiments	[159]
		Fruquintinib	Anti‐PD‐1 therapy	Microsatellite stability metastatic colorectal cancer	Clinical trial	[160]
		Ramucirumab	Paclitaxel	Advanced or metastatic gastric cancer	Clinical trial	[161]
		Anlotinib	None	Lewis lung carcinoma	Animal experiments	[162]
	S100A8/A9	Tasquinimod	Ixazomib, lenalidomide, dexamethasone	Multiple myeloma	Phase Ib/IIa clinical trial	[163]
	CXCL1	Metformin	None	Murine colon cancer	Animal experiments	[164]
	CXCR2	SB225002	Withaferin A, α‐PD‐1	Liver tumors	Animal experiments	[165]
	CXCR1/2	SX‐682	Anti‐PD‐1 therapy	Pancreatic ductal adenocarcinoma	Animal experiments	[51]
	CCR5	Met‐CCL5	None	Breast cancer	Animal experiments	[166]
		Maraviroc	Pembrolizumab	Refractory mismatch repair proficient/microsatellite‐stable metastatic colorectal cancer	Phase I clinical trial	[167]
Promoting the differentiation of MDSCs into nonsuppressive immune cells	RAR/RXR	ATRA	DC‐p53 vaccine	Advanced‐stage small cell lung cancer	Phase II clinical trial	[168]
	TLR7/8	Resiquimod	None	Actinic keratosis	Clinical trial	[169]
Promoting the apoptosis of MDSCs	STAT3	Napabucasin (BBI608)	None	Refractory advanced colorectal cancer	Phase III clinical trial	[170]
		Danvatirsen (AZD9150)	None	Diffuse large B‐cell lymphoma	Phase Ib clinical trial	[171]
	HDAC	Fimepinostat (CUDC‐907)	None/rituximab	Relapsed/refractory diffuse large B‐cell lymphoma	Phase I clinical trial	[172]
Inhibiting MDSC metabolism	mTOR	Rapamycin (Sirolimus)	None	Malignant perivascular epithelioid cell tumors	Phase II clinical trial	[173]
	CPT1	Etomoxir	None	Lewis lung carcinoma, MCA‐38 colon adenocarcinoma	Animal experiments	[174]
	IDO	Epacadostat (INCB024360), navoximod (GDC‐0919, NLG‐919), PF‐06840003, BGB‐5777, linrodostat (BMS‐986205)	None	Murine tumor model	Animal experiments	[175]
Inhibiting the immunosuppressive function of MDSCs	VDR	1,25(OH)_2_D3	None	Head and neck squamous cell carcinoma	Clinical trial	[176]
	PDE5	Tadalafil	None	Metastatic melanoma	Clinical trial	[177]
	COX‐2	Celecoxib	None	Murine mesothelioma tumors	Animal experiments	[178]
			Fluorouracil, leucovorin, oxaliplatin	Colon cancer	Phase III clinical trial	[179]

*Abbreviations*: 1,25(OH)2D3, 1,25‐dihydroxyvitamin D3; ATRA, all‐trans retinoic acid; CCR, G protein‐coupled C‐chemokine receptors; COX, cyclooxygenase; CPT1, carnitine palmitoyltransferase 1; CXCL1, chemokine; CXCR1/2, chemokine receptors; HDAC, histone deacetylase; IDO, indoleamine 2,3‐dioxygenase; Met‐CCL5, a CCR5 antagonist; mTOR, mammalian target of rapamycin; PDE5, phosphodiesterase‐5; RAR, retinoic acid receptor; RXR, retinoid X receptor; S100A8/A9, calprotectin; SB225002, a CXCR2 inhibitor; STAT3, signal transducer and activator of transcription 3; SX‐682, a novel oral inhibitor of CXCR1/2; TLR, Toll‐like receptor; VDR, vitamin D receptor; VEGF, vascular endothelial growth factor.

### 5.1 Inhibiting MDSC Proliferation and Recruitment

#### 5.1.1 VEGF Inhibitors

As previously mentioned, VEGF plays a significant role in the nonimmunological functions of MDSCs, such as inducing the EMT, tumor angiogenesis, and the formation of premetastatic niches, making it a promising therapeutic target for limiting MDSC accumulation and enhancing antitumor immunity.

Bevacizumab, a monoclonal antibody that specifically inhibits VEGF, has been used in clinical practice for various malignancies, including metastatic colorectal cancer, NSCLC, and hepatocellular carcinoma [180]. Clinical studies have shown that bevacizumab can significantly reduce the percentage of the PMN‐MDSC subpopulation in patients with cancer, particularly in NSCLC patients [158]. For example, in patients treated with clinical regimens containing bevacizumab, a reduction in PMN‐MDSCs was observed compared with those receiving standard chemotherapy without bevacizumab [158]. These findings suggest that VEGF inhibition can disrupt MDSC‐mediated immunosuppression, making the tumor microenvironment more conducive to immune system activity.

Endostar, a recombinant human endostatin, and fruquintinib, both VEGF receptor (VEGFR) inhibitors, also show promise in reducing MDSC accumulation [159, 160]. In a 2020 study using a Lewis lung cancer (LLC) mouse model, the combination of Endostar and anti‐PD‐1 therapy not only significantly suppressed tumor growth but also reduced MDSC infiltration within the tumor site [159], indicating the potential of combinations of antiangiogenic agents and ICIs. Fruquintinib, another potent VEGFR inhibitor, has produced similar effects in clinical trials. A 2024 study revealed that when combined with PD‐1 inhibitors, fruquintinib markedly reduces PMN‐MDSC levels in patients, suggesting a synergistic effect on increasing the efficacy of immunotherapies [160].

Ramucirumab, a monoclonal antibody targeting VEGFR2, is commonly used to treat gastric cancer and NSCLC. A 2020 clinical trial that evaluated ramucirumab combined with paclitaxel reported a general reduction in PMN‐MDSCs after two cycles of treatment. However, patients who progressed after the second cycle often exhibited an increase in MDSC levels, with the accumulation of these cells correlating with poorer clinical outcomes [161]. This finding highlights the complexity of MDSC dynamics in the therapeutic response and underscores the need for continuous monitoring of MDSC populations as potential biomarkers for treatment efficacy and resistance.

Anlotinib, a multitarget tyrosine kinase inhibitor that blocks VEGFR, platelet‐derived growth factor receptor, fibroblast growth factor receptor (FGFR), and stem cell growth factor receptor (C‐KIT) kinases, also exerts antiangiogenic effects and has shown efficacy in various cancers [181]. In a 2023 study using the LLC mouse model, anlotinib significantly reduced M‐MDSC levels but did not significantly affect the PMN‐MDSC population [162]. This observation suggests that while VEGFR inhibitors such as anlotinib can modulate certain MDSC subsets, their effects may vary depending on the specific tumor model and MDSC subpopulation, requiring further investigation into the selective targeting of MDSC populations.

#### 5.1.2 S100A8/A9 and CD300ld

S100A8 and S100A9, small‐molecule calcium‐binding proteins, play pivotal roles in tumor progression. MDSCs coexpress S100A8/A9 proteins and receptors for advanced glycation end products, forming a feedback loop that promotes MDSC migration via the NF‐κB signaling pathway [13]. Recent findings from a team at Fudan University revealed that CD300ld is an upstream regulator of S100A8/A9. CD300ld induces STAT3 phosphorylation, subsequently upregulating S100A8 and S100A9 expression and ultimately mediating the migration and recruitment of PMN‐MDSCs [54].

Tasquinimod is an inhibitor of S100A8/A9. In tumor‐bearing mice, both tasquinimod treatment and CD300ld gene knockout inhibited PMN‐MDSC migration and reduced immunosuppressive effects, suggesting that drugs targeting CD300ld could exhibit comparable antitumor efficacy to those targeting S100A8/S100A9 [54]. In addition, clinical trials of tasquinimod for the treatment of multiple myeloma are ongoing (NCT04405167) [163].

#### 5.1.3 Inhibition of CXCL1 Expression

##### 5.1.3.1 Metformin

Metformin, a classic antidiabetic drug, is widely used to treat type II diabetes. Recent studies have shown that metformin can block the accumulation of PMN‐MDSCs through the AMPK/DACH1/CXCL1 axis [182]. Experiments in mouse models have demonstrated that, compared with control groups, tumor‐bearing mice treated with metformin presented a significantly reduced tumor mass and volume. Thus, metformin treatment represents a viable research direction for cancer therapy, particularly for patients with concurrent diabetes and cancer [164].

##### 5.1.3.2 Targeting Cysteine‐Rich Intestinal Protein 1

Cysteine‐rich intestinal protein 1 (CRIP1) is a key oncoprotein. Studies have shown that knocking down the CRIP1 gene in pancreatic tumor cells significantly downregulates the expression of CXCL1 and CXCL5. These findings suggest that CRIP1 plays a role in inducing the expression of these chemokines. Since MDSC migration relies primarily on the expression of the chemokine receptors CXCR1/2 and their homologous ligands CXCL1 and CXCL5, CRIP1 may influence MDSC migration through its effects on CXCL1 and CXCL5 expression.

Researchers have utilized patient‐derived xenograft tumor models to establish a pancreatic ductal adenocarcinoma model and then injected human peripheral blood mononuclear cells via the tail vein. The results revealed increased recruitment of MDSCs in samples expressing CRIP1, suggesting that targeting CRIP1 could be a viable therapeutic approach for cancer. Unfortunately, no specific drugs targeting CRIP1 have yet been developed in recent years, leaving this therapeutic strategy to be explored further [51].

#### 5.1.4 CXCR2 Blockade

The ligands for the CXCR2 receptor primarily include CXCL1, CXCL2, and CXCL5, which are all important chemokines of MDSCs. An analysis of CXCR2 expression in MDSCs revealed that nearly all circulating PMN‐MDSCs and 40% of bone marrow PMN‐MDSCs express CXCR2, suggesting a primary association of CXCR2 with the accumulation of PMN‐MDSCs [183].

A significant difference in the response to anti‐PD‐1 therapy was observed between wild‐type and CXCR2‐deficient tumor‐bearing mice. PD‐1 blockade was ineffective in wild‐type tumor‐bearing mice, whereas a significant reduction in tumor growth was observed in CXCR2‐deficient mice [184]. Additionally, the use of SB225002 (a CXCR2 inhibitor) in conjunction with Withaferin A (a natural anticancer agent that induces ferroptosis through lipid peroxidation in vivo) and α‐PD‐1 (an anti‐PD‐1 antibody) to block MDSC infiltration in tumor‐bearing mice led to increased survival rates [165].

SX‐682, a novel oral inhibitor of CXCR1/2, blocks the recruitment of tumor MDSCs and enhances T‐cell activation. In animal studies, the combination of anti‐PD‐L1 therapy with SX‐682 induced an increase in CD8^+^ T‐cell infiltration and effective antitumor activity in tumor‐bearing mice with high CRIP1 expression [51]. Related clinical trials are ongoing, and the results are highly anticipated.

#### 5.1.5 CCR5 Antagonists

Chemokines are known to regulate the trafficking of lymphocytes and myeloid cells through interactions with specific transmembrane, G protein‐coupled C‐chemokine receptors (CCRs). Current research indicates that CCR5 is associated with the proliferation and recruitment of MDSCs. Experiments have shown an increased frequency of CCR5‐expressing MDSCs in melanoma lesions compared with their frequency in the bone marrow and peripheral blood; furthermore, the expression levels of CCR5 on MDSCs also increase during tumor progression [185]. In a mouse model of breast cancer, treatment with Met‐CCL5 (a CCR5 antagonist) for 5 continuous weeks resulted in significant reductions in tumor volume and weight compared with those of control tumors, indicating the activity of Met‐CCL5 against established tumors [166].

Maraviroc is an orally administered CCR5 antagonist previously used to treat HIV infection. In a phase I clinical trial completed in 2020 (NCT03274804), the team aimed to explore the safety and efficacy of treatment with pembrolizumab (a PD‐1 inhibitor) and maraviroc for refractory mismatch repair‐proficient/microsatellite stable metastatic colorectal cancer. However, the study has not yet determined the role of maraviroc in reducing the number of MDSCs, and thus further investigation into this topic is warranted [167].

### 5.2 Promotion of MDSC Differentiation into Nonsuppressive Immune Cells

#### 5.2.1 All‐Trans Retinoic Acid

All‐trans retinoic acid (ATRA), an intermediate metabolite of vitamin A in the body and a member of the retinoid molecular family, has potent regulatory effects on cell proliferation, the induction of differentiation, and apoptosis. Research has shown that ATRA, by activating extracellular signal‐regulated kinases 1/2, upregulates the expression of glutathione synthetase, thereby generating high levels of glutathione (GSH). GSH, by neutralizing accumulated intracellular ROS, mediates the differentiation of MDSCs into Mφs and DCs, thereby reducing the immunosuppressive effect of MDSCs on the organism [186].

A clinical trial conducted in 2013 involving patients with advanced‐stage small cell lung cancer showed that patients treated with ATRA had a more than twofold reduction in MDSC levels, showcasing the efficacy of ATRA treatment [168].

#### 5.2.2 Toll‐Like Receptor Agonists

In mammals, Toll‐like receptor (TLR)‐mediated innate immune mechanisms are associated with defenses against fungal, bacterial, and viral infections. With advancements in research, some teams have discovered the indispensable role of TLRs in promoting MDSC differentiation. Currently, studies have shown that MDSCs isolated from mice harboring breast cancer cells cultured with resiquimod (a TLR7/8 agonist) for 5 days exhibit increased differentiation into Mφs and DCs. Additionally, the differentiated MDSCs lose their suppressive activity against T cells [187].

Resiquimod has previously been studied in the field of vaccine adjuvants. A 2019 clinical trial demonstrated its effectiveness in treating actinic keratosis (a precancerous skin condition), although it did not specify whether this effect was related to the pro‐differentiation effect on MDSCs. Further research is needed to explore this potential relationship [169].

### 5.3 Promotion of MDSC Apoptosis

#### 5.3.1 STAT3 inhibitors

STAT3 is a cytoplasmic transcription factor that becomes hyperactivated in most human cancers and is often associated with a poor clinical prognosis [188]. Studies have shown that in mouse models, STAT3 inhibition can promote the apoptosis of liver‐associated MDSCs (L‐MDSCs). The proposed mechanism is that the inhibition of STAT3 can upregulate the expression of Fas (a cell death receptor), which, upon binding to its ligand FasL, promotes the formation of homodimers of the Bax protein, leading to mitochondrial morphological changes and the release of cytochrome C, thereby inducing MDSC apoptosis [189].

Imatinib, a STAT3 inhibitor synthesized in 1992, primarily exerts its inhibitory effect on STAT3 by inhibiting upstream Src kinases and has been extensively applied in clinical practice. Moreover, napabucasin (BBI608), a small‐molecule STAT3 inhibitor, not only completely eradicated the immunosuppressive activity of murine MDSCs and human M‐MDSCs in mouse melanoma models, significantly improving the survival rate of treated mice, but also represents the only drug to enter phase III clinical trials (NCT01830621). The clinical trial showed that only patients with pSTAT3‐positive tumors in the BBI608 group experienced longer survival than did those in the placebo group; unfortunately, no difference in OS was observed between the groups [170, 190]. Danvatirsen (AZD9150) is a specific antisense oligonucleotide (ASO) inhibitor that targets STAT3. A phase Ib clinical trial (NCT01563302) conducted in 2018 indicated that AZD9150 treatment of 27 patients with diffuse large B‐cell lymphoma (DLBCL) significantly reduced PMN‐MDSC levels, with patients showing remission and good tolerance to the treatment. Moreover, studies combining AZD9150 with checkpoint immunotherapy are currently underway, showing promising potential for improved clinical outcomes [171].

#### 5.3.2 Histone Deacetylase Inhibitors

Histone deacetylases (HDACs) are a class of proteases that play crucial roles in modifying the chromosomal structure and regulating gene expression, and histone deacetylase inhibitors (HDACis) have shown excellent anticancer effects and have been extensively researched. Studies of mouse models of breast cancer have confirmed that HDACis increase MDSC apoptosis, thereby reducing the accumulation of MDSCs in tumors. However, this apoptotic effect could be abrogated by N‐acetylcysteine (an ROS scavenger), suggesting that HDACis likely induce MDSC apoptosis by increasing intracellular ROS levels in MDSCs [191].

HDACis are widely used in clinical practice to treat hematologic malignancies. A phase I clinical trial (NCT01742988) involving the novel HDAC and PI3K inhibitor fimepinostat (CUDC‐907) has shown that it has antitumor activity in DLBCL patients with tolerable safety, and we look forward to the continued development of HDACis such as CUDC‐907 in these populations with substantial unmet needs [172].

#### 5.3.3 Other Therapeutic Approaches

Doxorubicin and 5‐fluorouracil (5‐FU) are classic chemotherapeutic agents that inhibit tumor cell growth by interfering with DNA synthesis. In addition to their effects on tumor cells, these agents are also effective at inducing apoptosis in MDSCs. In a mouse model of breast cancer, doxorubicin selectively depleted PMN‐MDSCs but had little effect on M‐MDSCs; in a thymoma mouse model, 5‐FU significantly depleted both PMN‐MDSCs and M‐MDSCs [192, 193].

Additionally, paclitaxel, another traditional chemotherapeutic agent, inhibits microtubule depolymerization, thereby preventing spindle formation and inhibiting mitosis in tumor cells, promoting tumor cell death as a natural substance with anticancer activity. Moreover, low concentrations of paclitaxel also promote the differentiation of MDSCs into DCs [194]. Clinical trials have shown that for women with breast cancer, weekly paclitaxel treatment after standard adjuvant chemotherapy with doxorubicin and cyclophosphamide improved both disease‐free and OS [195].

Interestingly, curcumin (CUR), a plant pigment with high antioxidant capacity that is nontoxic to humans, can also reduce MDSC levels. Studies have shown that CUR selectively diminishes the proportion of PMN‐MDSCs in tumor‐bearing mice [196]. In addition, in 2024, a team designed an ROS/GSH dual‐responsive drug conveyance platform (CUR/miR155@DssD‐Hb NPs) to codeliver CUR and microRNA‐155 (miR‐155, which can induce MDSC accumulation in tumors). Experiments have shown that tumors treated with CUR/miR155@DssD‐Hb NPs exhibit a reduction in MDSC recruitment, possibly related to the depletion of MDSCs within the tumor, revealing the potential of CUR in reshaping the immunosuppressive TME [197].

### 5.4 Inhibiting MDSC Metabolism

#### 5.4.1 Targeting the Glycolysis Pathway

Current research indicates that low‐dose treatment with the hexokinase inhibitor 2‐deoxy‐d‐glucose (2‐DG) can lead to a significant reduction in the degree of differentiation of bone marrow cells toward M‐MDSCs, making 2‐DG a viable approach for inhibiting MDSCs.

Furthermore, the PI3K/AKT pathway, a classic insulin signaling pathway, plays a role in promoting cellular glucose uptake and utilization. Experiments have confirmed that the AS160 protein, which is downstream of the PI3K/AKT pathway, regulates glucose uptake [198]. Another protein involved in the PI3K/AKT pathway, mTOR, can also regulate AKT activity, thereby inhibiting glycolysis in MDSCs. Studies have shown that the absence of mTOR in bone marrow cells can selectively block the differentiation of the M‐MDSC subset in allogeneic skin graft mice [199]. Consequently, mTOR inhibitors such as rapamycin (sirolimus) can effectively reduce glycolysis, immunosuppressive activity, and the percentage of tumor‐infiltrating M‐MDSCs in tumor‐bearing mice [13, 200]. A phase II clinical trial conducted in 2021 (NCT02494570) demonstrated the significant efficacy and good tolerability of rapamycin in the treatment of patients with malignant peripheral nerve sheath tumors [173].

Additionally, in colorectal cancer cells, the activated PI3K/AKT pathway was recently reported to upregulate HIF‐1α expression in 5‐FU‐resistant colorectal cancer cells, thereby increasing the expression of membrane transport proteins to increase glucose flux and enhance glycolysis. Thus, targeting glycolysis via the PI3K/AKT pathway may become a future direction for oncological drug development [201].

#### 5.4.2 Targeting the Fatty Acid Oxidation Pathway

Fatty acid oxidation (FAO) is a crucial energy metabolism pathway for tumor‐infiltrating MDSCs (T‐MDSCs). In mouse tumor models, daily injection of etomoxir, a specific inhibitor of carnitine palmitoyltransferase 1 (CPT1, the first rate‐limiting enzyme in the FAO pathway), significantly reduced the overall metabolic pathway of T‐MDSCs. The glycolysis pathway could not compensate in this context, thereby reducing T‐MDSC tolerance. Concurrently, with the inhibition of FAO, the immunosuppressive function of T‐MDSCs also decreases, impairing their ability to block T‐cell proliferation and IFN‐γ production. Unfortunately, related clinical studies are scarce, and etomoxir is presumably still in the experimental research stage [174].

#### 5.4.3 Targeting the Tryptophan Pathway

IDO suppresses effector T‐cell activation via tryptophan depletion while metabolizing tryptophan into kynurenine to activate AHR, thereby inducing the secretion of cytokines such as IL‐10 and IL‐21, which promote Treg differentiation and activation [202]. Treg activation enhances the suppression of the immune system, facilitating the production and recruitment of MDSCs.

Clinical trials have shown effective antitumor activity and the restoration or activation of cancer immune surveillance functions with IDO inhibitors such as epacadostat (INCB024360), navoximod (GDC‐0919, NLG‐919), PF‐06840003, BGB‐5777, and linrodostat (BMS‐986205) in oncological models [175].

### 5.5 Inhibiting the Immunosuppressive Function of MDSCs

#### 5.5.1 1,25‐Dihydroxyvitamin D

1,25‐Dihydroxyvitamin D (1,25(OH)_2_D) includes 1,25(OH)_2_D2 and 1,25(OH)_2_D3, which are the main forms of vitamin D2 (ergocalciferol) and D3 (cholecalciferol) produced after metabolism by the liver and kidneys, respectively. Studies have shown that treating tumor‐associated MDSC‐like cells with 1,25(OH)_2_D significantly reduces their ability to suppress T cells [203]. A clinical study published in 2010 revealed that patients with head and neck squamous cell carcinoma treated with 1,25(OH)_2_D3 had significantly increased levels of CD4^+^ and CD8^+^ T cells in their tissues and a longer time to tumor recurrence [176]. Unfortunately, recent drug development efforts in this area have not yet yielded successful clinical applications. Further research and development are needed to address these challenges.

#### 5.5.2 Phosphodiesterase 5 Inhibitors

Phosphodiesterase‐5 (PDE5) inhibitors, such as tadalafil and sildenafil, are currently used in clinical practice for nonmalignant conditions. Studies of mouse models have shown that sildenafil can reduce the expression and enzymatic activity of Arg1 and nitric oxide synthase‐2 (NOS2). Since Arg1 and NOS2 are key enzymes in the immunosuppressive pathways of MDSCs, PDE5 inhibitors can suppress the immunosuppressive function of MDSCs [204]. Clinical trials have demonstrated that tadalafil is effective, safe and well tolerated when used to treat patients with metastatic melanoma. These findings underscore the potential of PDE5 inhibitors for targeting MDSCs in treatment [177].

#### 5.5.3 COX‐2/PGE2 Axis Inhibitors

PGE2 is a physiologically active fatty acid that is often involved in the regulation of inflammation, angiogenesis, and tumor progression. Research has shown that PGE2 is also involved in regulating the primary immunosuppressive functions of MDSCs. Since COX‐2 is the key enzyme in the synthesis of PGE2, COX‐2‐targeted therapy and PGE2‐targeted therapy can serve as similar immunomodulatory treatments [92].

Experiments have shown that Mo‐MDSCs derived from human melanomas inhibit NK cell activity by producing TGF‐β. Similarly, monocytes treated with PGE2 inhibit NK cell activity through the production of TGF‐β, similar to Mo‐MDSCs [205]. Therefore, COX‐2/PGE2 axis inhibitors can suppress PGE2 production, thereby reducing the generation of Mo‐MDSCs and weakening the immunosuppressive effects of Mo‐MDSCs. In animal studies, tumor‐bearing mice receiving a celecoxib (a COX‐2 inhibitor)‐containing diet had a significantly lower absolute number of MDSCs than did tumor‐bearing mice receiving a control diet [178]. Unfortunately, a clinical study conducted in 2021 on the use of celecoxib for the treatment of stage III colon cancer (NCT01150045) showed that, compared with the placebo group, a significant improvement in DFS was not observed in the celecoxib treatment group, indicating that the efficacy of this therapy remains to be explored [179].

## 6 Emerging Technologies for Targeting MDSCs

**FIGURE 5:**
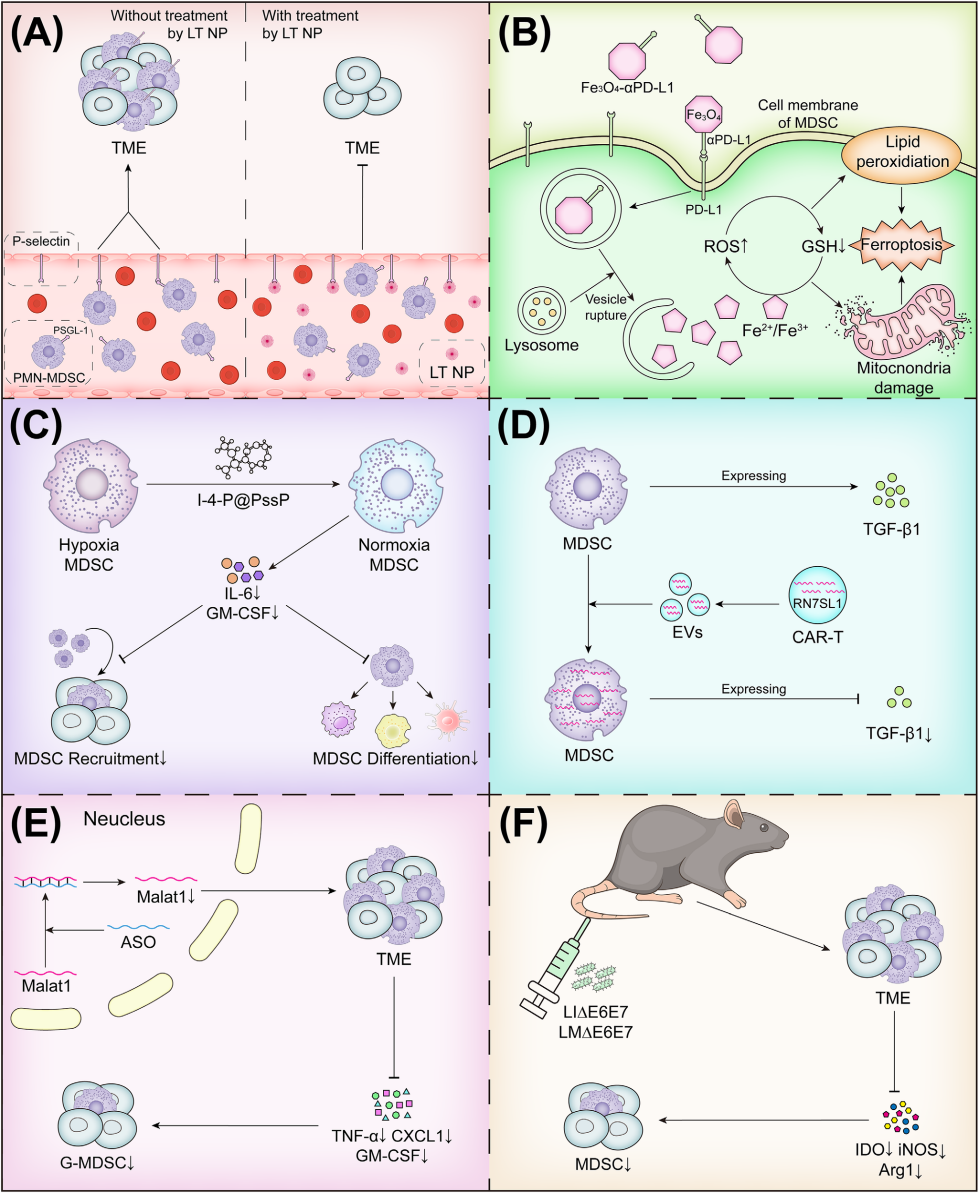
Emerging technologies for targeting MDSCs. (A–C) Nanotechnology‐mediated MDSC modulation. (A) Low‐molecular‐weight heparin‐tocopherol succinate nanoparticle (LMWH‐TOS nanoparticle, LT NP) effectively inhibit PMN‐MDSCs transmigration from vasculature to tumor tissues during early recruitment phase. (B) PD‐L1‐targeting ultrasmall iron oxide nanoprobes (Fe_3_O_4_–αPD‐L1) disrupt intracellular redox balance in MDSCs, which ultimately induces ferroptosis of MDSCs. (C) The dual‐oxygen supply nanoplatform (IR‐1048–4‐MU–PFOB@mPEG‐SS‐PLGA, I‐4‐P@PssP) alleviate tumor hypoxia, leading to reduced MDSC infiltration. (D and E) Gene editing‐based MDSC suppression. (D) RN7SL1‐enriched extracellular vesicles derived from CAR‐T cells selectively eliminate MDSCs while blocking TGF‐β‐mediated immunosuppressive pathways. (E) Malat1‐targeting antisense oligonucleotides (ASOs) downregulate chemokines like CXCL1, GM‐CSF, and TNF‐α secretion in the TME, thereby impair PMN‐MDSC recruitment, development, and immunosuppression. (F) Vaccine‐based MDSC regulation. Neoantigen vaccines (LM∆E6E7 and LI∆E6E7) significantly downregulate immunosuppressive enzymes like Arg1, IDO, and iNOS, effectively weakening the immunosuppressive function of MDSCs in tumor sites. *Abbreviations*: LT NP, micellar nanoparticle composed of low‐molecular‐weight heparin‐tocopherol succinate; PSGL‐1, P‐selectin glycoprotein ligand‐1; Fe_3_O_4_–αPD‐L1, ultrasmall paramagnetic iron oxide nanoprobe targeting PD‐L1; I‐4‐P@PssP, dual‐oxygen supply nanoplatform; RN7SL1, an endogenous RNA that activates RIG‐I/MDA5 signaling; Malat1, metastasis‐associated lung adenocarcinoma transcript; ASO, gapmer antisense oligonucleotide; LI∆E6E7, cervical cancer vaccine constructed by *Listeria ivanovii*; LM∆E6E7, cervical cancer vaccine constructed by *Listeria monocytogenes*.

Compared with traditional targeted therapeutic strategies for MDSCs, which often cause significant side effects due to low drug specificity, emerging treatment technologies that target MDSCs tend to exhibit greater specificity, making them promising candidates for further research and development. Brief summaries of recent research findings on three emerging technologies are provided below (Figure 5).

### 6.1 Nanotechnologies

In 2020, a team developed a micellar nanoparticle composed of low‐molecular‐weight heparin‐tocopherol succinate (LMWH‐TOS nanoparticle, LT NP), which, through its hydrophilic segment LMWH, can inhibit the adhesion between endothelial cells and PMN‐MDSCs mediated by P‐selectin/PSGL‐1; thus, it can block the early recruitment of PMN‐MDSCs from blood vessels into tissues [206, 207]. Additionally, in 2024, Wang team [208] developed a PD‐L1‐targeted ultrasmall paramagnetic iron oxide nanoprobe (Fe_3_O_4_–αPD‐L1), which enters MDSCs by binding to the highly expressed PD‐L1 receptor on MDSCs after radiotherapy and eventually accumulates in the lysosomes to release Fe^2+^/Fe^3+^, inducing a large amount of ROS production, causing an oxidative stress imbalance in MDSCs, and ultimately inducing ferroptosis. Recently, IR‐1048, a near‐infrared‐II (NIR‐II) fluorescent molecule, 4‐methylumbelliform one (4‐MU), and perfluorooctane bromide (PFOB) were coloaded onto a GSH‐responsive amphiphilic polymer (mPEG‐SS‐PLGA) to construct a dual‐oxygen supply nanoplatform (IR‐1048–4‐MU–PFOB@mPEG‐SS‐PLGA, I‐4‐P@PssP), which may alleviate chronic hypoxia in the TME to inhibit MDSC infiltration, thereby reducing the tumor volume and prolonging the survival of mice. This result highlights the importance of eliminating intratumoral MDSCs in tumor therapy [209].

### 6.2 Gene Editing Techniques

In 2021, a team delivered RN7SL1 (an endogenous RNA that activates RIG‐I/MDA5 signaling) through CAR‐T cells. RN7SL1 can be selectively transferred to immune cells via EVs and limit the accumulation of MDSCs, reducing their expression of TGF‐β1 [210, 211]. Metastasis‐associated lung adenocarcinoma transcript (Malat1) is an abundant long noncoding RNA that is upregulated in various cancers. In 2023, a team knocked down Malat1 RNA expression using gapmer ASOs and found that the levels of the chemokines CXCL1, GM‐CSF, and TNF‐α, which support MDSC recruitment, development, and immune suppression in the TME, were reduced, decreasing the number of PMN‐MDSCs in the tumor [212].

### 6.3 Tumor Vaccine Techniques

*Listeria monocytogenes* (LM) is a commonly used bacterial vector for therapeutic cancer vaccine. In 2024, researchers developed two novel cervical cancer vaccine candidates, LM∆E6E7 and LI∆E6E7, using attenuated *Listeria monocytogenes* (LM∆) and attenuated *Listeria ivanovii* (LI∆), both of which lack the actA and plcB virulence genes. These candidates stably express the HPV‐16 E6 and E7 fusion antigen, and were shown to inhibit the enrichment and infiltration of MDSCs at tumor sites in cervical cancer‐bearing mice. Additionally, they weakened the immunosuppressive function of MDSCs by downregulating the expression of Arg1, IDO, and iNOS [213].

## 7 Conclusions and Perspectives

In conclusion, the evolving understanding of MDSCs in cancer immunology underscores their central role in tumor progression. Despite significant advances in characterizing their function, many aspects of MDSC biology, including the precise mechanisms underlying their recruitment, differentiation, and immunosuppression, remain unclear. The heterogeneity and plasticity of these cells present ongoing challenges for achieving specificity and effectiveness in targeted therapeutic strategies while also offering promising avenues for developing more precise and effective interventions.

Recent breakthroughs, such as single‐cell RNA sequencing and spatial transcriptomics, have provided unprecedented insights into the diversity of MDSC subsets within the tumor microenvironment [214, 215, 216, 217]. These technologies are poised to reveal molecular signatures that distinguish functionally distinct MDSC populations, providing potential tools for the development of novel biomarkers and more personalized therapies.

A critical area for therapeutic exploration lies in the interactions between MDSCs and immune checkpoint pathways, such as PD‐1/PD‐L1. As resistance to immune checkpoint blockade becomes an increasing challenge, combination therapies that simultaneously target MDSCs and immune checkpoints hold considerable promise. For example, bispecific antibodies or combination regimens that modulate MDSC activity while increasing checkpoint signaling might improve the efficacy of current immunotherapies, especially in cancers that are resistant to single agents [218].

Metabolic reprogramming has also emerged as a central mechanism driving MDSC immunosuppressive activity. Targeting key metabolic pathways, such as arginine metabolism and FAO, could provide therapeutic opportunities to selectively inhibit MDSCs without compromising other immune cells [174, 175]. Further investigations into metabolic regulators such as mTOR and HIF‐1α may reveal new strategies to complement existing immunotherapies and enhance antitumor responses.

The role of exosome‐mediated communication between MDSCs and other immune cells offers another frontier for intervention. Exosomes derived from MDSCs are known to carry immunosuppressive molecules and noncoding RNAs that can reprogram the immune environment to favor tumor growth [116, 118]. Strategies targeting these exosomes or blocking their interactions with immune cells could provide a novel approach to restore antitumor immunity.

Additionally, the influence of the microbiome on MDSC function has garnered increasing attention. Accumulating evidence suggests that microbial communities, particularly those in the gut, can modulate MDSC activity and shape the tumor immune response [91]. This microbiome–MDSC axis represents a new layer of complexity that could be harnessed for therapeutic benefits. Future studies should explore how microbiome modulation, such as through the use of probiotics or prebiotics, can influence MDSC function and increase the efficacy of cancer immunotherapies.

The integration of novel technologies is poised to revolutionize MDSC‐targeted therapies. Approaches such as nanotechnology and gene editing techniques show great promise in overcoming the limitations of traditional therapies by increasing specificity and efficiency at the molecular level [208, 210–212]. For example, gene expression modulation has successfully reduced MDSC numbers in preclinical models, highlighting the potential of gene therapy in this context. Furthermore, tumor vaccine technologies targeting MDSCs may complement current strategies by alleviating MDSC‐mediated suppression and enhancing the antitumor response.

In the near future, a concerted effort across clinical and translational research will be crucial to advance MDSC‐targeted therapies. The integration of emerging technologies, such as gene editing, nanomedicine, and immunotherapy, holds great promise for overcoming the barriers posed by MDSCs in cancer treatment. Ongoing and future clinical trials are expected to provide insights into the efficacy of these targeted therapies, particularly in neoadjuvant settings or as first‐line treatments. Ultimately, overcoming the immunosuppressive function of MDSCs could represent a major breakthrough in cancer therapy, offering new hope for patients with difficult‐to‐treat cancers and improving the overall outcomes of current treatments.

## Author Contributions

All authors contributed to manuscript writing. Tianying Hu conducted literature searches, drafted the original manuscript, and reviewed the figures. Jianxue Zhai drafted the original manuscript and provided critical comments and suggestions. Zhanda Yang, Jiajia Peng, and Chuxuan Wang analyzed the documents and designed table/figures. Xinyao Liu, Yawen Li, Jiaqi Yao, Fengxi Chen, Haixia Li, and Taixue An conducted literature searches and provided critical suggestions. Haifang Wang and Zongcai Liu conceived the review topic, designed the framework, and supervise revisions. All authors have read and approved the final manuscript.

## Conflicts of Interest

The authors declare no conflicts of interest.

## Ethics Statement

The authors have nothing to report.

## Data Availability

The datasets used or analyzed during the current study are available from the corresponding author on reasonable request.
